# Long-Term Exercise and Risk of Metabolic and Cardiac Diseases: The Erlangen Fitness and Prevention Study

**DOI:** 10.1155/2013/768431

**Published:** 2013-07-30

**Authors:** Wolfgang Kemmler, Simon von Stengel, Michael Bebenek, Willi A. Kalender

**Affiliations:** Institute of Medical Physics, University of Erlangen-Nuremberg, Henkestraße 91, 91052 Erlangen, Germany

## Abstract

In female subjects, ageing and the menopausal transition contribute to a rapid increase of metabolic and cardiac risk factors. Exercise may be an option to positively impact various risk factors prone to severe metabolic and cardiac diseases and events. This study was conducted to determine the long-term effect of a multipurpose exercise program on metabolic and cardiac risk scores in postmenopausal women. 137 osteopenic Caucasian females (55.4 ± 3.2 yrs), 1–8 years postmenopausal, were included in the study. Eighty-six subjects joined the exercise group (EG) and performed an intense multipurpose exercise program which was carefully supervised during the 12-year period, while 51 females maintained their habitual physical activity (CG). Main outcome measures were 10-year coronary heart disease risk (10 y CHD risk), metabolic syndrome *Z*-score (MetS Index), and 10-year myocardial infarction risk (10 y hard CHD risk). Significant between-group differences all in favor of the EG were determined for 10 y-CHD risk (EG: 2.65 ± 2.09% versus CG: 5.40 ± 3.30%; *P* = 0.001), MetS-Index (EG: −0.42 ± 1.03% versus CG: 1.61 ± 1.88; *P* = 0.001), and 10 y-hard-CHD risk (EG: 2.06 ± 1.17% versus CG: 3.26 ± 1.31%; *P* = 0.001). Although the nonrandomized design may prevent definite evidence, the intense multi-purpose exercise program determined the long-term efficacy and feasibility of an exercise program to significantly impact metabolic and cardiac risk scores in postmenopausal women. This trial is registered with ClinicalTrials.gov NCT01177761.

## 1. Introduction

Multimorbidity of the elderly is an increasing problem in the western world. With respect to community dwelling subjects, in Germany, two thirds of the female population, 55–69 years old, showed two to four diseases [1]. Beside musculoskeletal problems, metabolic and cardiac diseases largely contribute to the high morbidity of our elderly population [2, 3]. Due to the unfavorable demographic development this dilemma will not only increasingly stress our health systems but also impact the subject's quality of life and independence [4]. Thus, effective strategies to prevent diseases largely related with increasing age are of high priority. Unlike dedicated pharmaceutical agents, exercise represents a complex agent that affects most, if not all, of the relevant risk factors and diseases of the elderly [5–7]. However, although evidence for the positive effect of exercise per se on various risk factors and diseases is quite convincing [5–7], multiple purpose exercise protocols that focus on more than one or two relevant risk factors and diseases of the elderly are scarce. Further, most exercise trials were rather short, rarely exceeding 6 months. Moreover, even when these protocols were effective during the first months of exercise, this does not guarantee general effectiveness. Consequently, the consistent long-term effects of exercise on various endpoints still have to be determined. 

To adequately address these issues, the goal of the Erlangen Fitness and Prevention Study (EFOPS) is to validate a long-term general purpose exercise program with reasonable training volume that could be adopted by other health sports institutions or organizations. In this paper the authors focus on the effect of this ongoing 12-year exercise program on metabolic and cardiac risk scores. The hypothesis was tested that changes among the exercisers for Framingham based 10-year CHD risk [8], metabolic syndrome *Z*-score [9, 10], and 10-year hard CHD risk (i.e., risk of myocardial infarction or coronary death) [10] were significantly more favorable compared with nontraining controls.

## 2. Materials and Methods

The Erlangen Fitness and Osteoporosis Prevention Study (EFOPS) is an ongoing controlled exercise trial that determines the long-term effects of exercise on various risk factors with a particular focus on fractures and bone mineral density in (early) postmenopausal women with osteopenia. The study protocol was approved by the ethics committee of the Friedrich-Alexander University of Erlangen-Nuremberg (Ethikantrag 905 and 4209) and the Bundesamt für Strahlenschutz (S9108-202/97/1). The EFOPS study was initiated and headed by the Institute of Medical Physics, University of Erlangen, Germany. The study reported here started in October 1998. The 12-year follow-up assessments were performed in September/October, 2010, and corresponding analyses were conducted up to April, 2011. All study participants gave written informed consent.

### 2.1. Subjects

**Figure 1:**
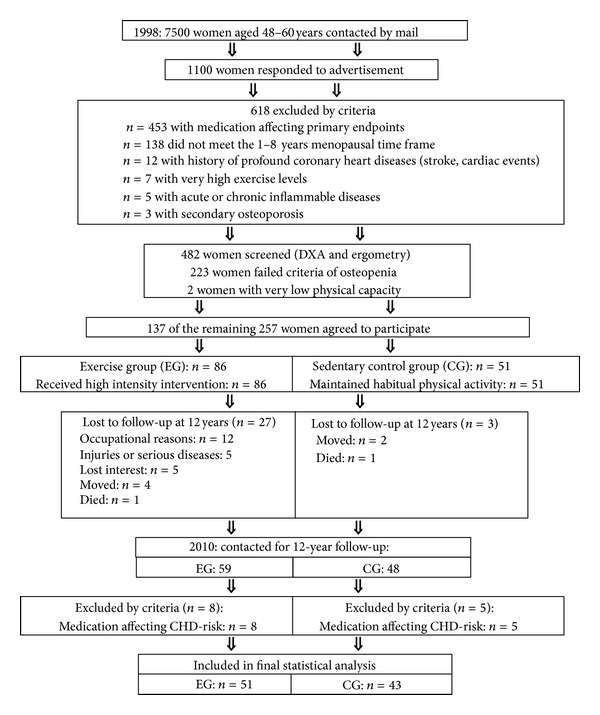
Extended flow chart of the EFOPS study.

Figure 1 gives the participant flow of the EFOPS study. Briefly, 1,100 Caucasian women 48–60 years old responded to personal mails and were checked for eligibility. In a first step 618 females were excluded by interview for reasons given in the flow chart (Figure 1). The remaining subjects were invited to check further eligibility criteria. 223 women did not meet the inclusion criteria of osteopenia and two subjects were excluded due to (very) low physical activity (<75 Watt) at cycle ergometry. Finally, 137 eligible women agreed to participate in the trial. Based on their own decision, 86 subjects participated in the exercise group (EG) and 51 subjects joined the control group (CG). The EG underwent the exercise program described later, while participants of the CG were requested to continue their normal lifestyle and habitual physical activity. During the 12 years of the study course, 27 subjects of the EG and 3 subjects of the CG were lost for reasons given in Figure 1. Baseline characteristic of the subgroups (EG, CG) did not differ between the cohort presented here and the initial cohort included in 1998. Of relevance, although all subjects of the EG that quit the program for the reasons given in Figure 1 were invited to the 12-year follow-up, but all subjects refused to attend the assessment.

Thus, in 2010, 59 subjects of the EG and 48 subjects of the CG were assessed within the 12-year follow-up. However, 8 subjects of the EG and 5 subjects of the CG were excluded from the statistical analysis by protocol due to medication directly affecting the primary and secondary endpoints. Thus, 51 subjects of the EG and 43 subjects of the CG were included in the 12-year follow-up analysis.  Table 1 shows baseline characteristics of the exercise and control group. No significant differences between both groups were determined.

### 2.2. Study Intervention

A block periodization scheme with 12 weeks of high intensity exercise specifically dedicated to muscle and bone was intermitted by 6 week periods of exercise with increased volume/lower intensity. High intensity blocks were structured into 3 cycles of 4 weeks using linear periodization. 

All group sessions were closely supervised by certified instructors who monitored and controlled compliance of the subjects. After the end of each 6 or 12 week period, training logs of the participants were analyzed to determine compliance and to check the rate of perceived exertion listed by the participants. Importantly, no sanctions were imposed on participants who did not regularly exercise at home in order to reduce potential motivations to cheat with the training logs. Apart from three weeks of holiday (Christmas, Easter) the group exercise program was rigorously maintained throughout the year. Total amount of exercise classes (2 sessions/week) and home training (2 sessions/week) averaged around 200 sessions per year (3.85 sessions/week). 

#### 2.2.1. Exercise Program

The complex exercise protocol of the EFOPS study was described in more details in earlier publications [11, 12]; thus only a summary will be given here.

Briefly, the program consists of two supervised group sessions (*≈*60 min) performed on nonconsecutive days of the week and two home training sessions (*≈*20–25 min). 

#### 2.2.2. Supervised Group Session

Generally, the group exercise sessions were structured into three main sequences: (1) 20–25 min of warmup/endurances exercises, (2) 3–5 min of jumping exercises, and (3) a 35–40 min resistance exercise training sequence. 

(1) During the endurance sequence 5–10 minutes of different running exercises and 10–15 minutes of low and high impact aerobic exercises with a progressively increasing amount of high impact exercises were conducted. Heart rates (HR) averaged 70%–85% HRmax (as assessed during stepwise treadmill test to a voluntary maximum) during this phase.

(2) The jumping exercise section started after 6 months of initial conditioning and was specifically dedicated to impact bone. After 2 min rope skipping exercise, 4 different jumping exercises with 1 set of 15 repetitions with 30 sec of rest between each exercise were performed. HR averaged between 65% and 90% HFmax during this section.

(3) The strength and power section consisted of one session/week on resistance machines (Techno Gym, Gambettola, Italy) and one session/week using isometric exercises, elastic bands, and free weights. During the session on machines 9–13 exercises affecting all the main muscle groups were performed. Twelve weeks of periodized high intensity resistance training (9-10 exercises with 1–4 sets and *≈*12–4 reps with 70%–92.5% 1 RM) were interleaved with 6-week transitional periods of lower intensity (*≈*55% 1 RM) but higher volume (13 exercises with 2-3 sets and 20–25 reps). Beside one 12-week “power” block per year with explosive concentric movements [13], time under tension was structured in a 2 s concentric—1 s isometric—2 sec eccentric movement mode. Initially individual resistance training plans of the subjects based on 1 RM tests. After five years of this procedure, load prescription given by the training plans was based on repetition number combined with the rate of perceived exertion which was assessed to be comparably effective [14].

During the second resistance training session/week 12–15 isometric exercises (2–4 sets, 6–10 sec) for trunk flexors/extensors, hip extensors/flexors, and leg abductors/adductors were performed with maximum effort followed by 20–30 s of active (stretching) rest. Additionally 3 elastic band exercises (2–4 sets and 15–20 reps.) dedicated to shoulder and upper back were carried out. Finally, three resistance exercises using free weights and weighted vests (squat/dead lift, one hand dumbbell rowing, and dumbbell chest press) were performed according to the periodized protocol described earlier. 

#### 2.2.3. Home Training Session

After a brief warmup (3 min of LI-Aerobic-routine) the nonsupervised home training consisted of 2-3 min of different rope skipping exercises, 6–8 isometric floor exercises, and 2 elastic band exercises with 2 sets each described earlier. Four to five stretching exercises were carried out during the corresponding rest periods. Each 12–24 weeks new home training routines replaced the existing protocols. 

In summary, total intensity of the present exercise protocol was rather high; however, it was not focused on complete exhaustion of the subjects, which was notably manifested by the approach of prescribing a submaximal number of repetitions per load.

### 2.3. Measurements

Measurements were carried out by research assistants blinded to the status of the participant. Further all baseline and follow-up tests described here were performed by the same assistants at the same time of day (±1 h). Testing procedures described later were performed at baseline and after years 1, 2, 3, 4, 5, and 12. However, primary endpoints of this contribution were retrospectively calculated for baseline and year 12.

#### 2.3.1. Primary Outcome Measures

The primary outcome measures of this study were “10-year CHD risk” according to Wilson et al. [8] and the MetS-*Z*-Score proposed by Johnson et al. [9] based on the NCEP Adult Treatment Panel (ATP) III MetS-Definition [10]. Secondary outcome was “10-year hard CHD risk” (i.e., risk of myocardial infarction and cardiac death; [10]).

#### 2.3.2. Anthropometry

Body weight and body height were always determined with the same standardized devices and procedures. Body composition was measured using the bioimpedance technique (Tanita BF 305, Tanita, Japan). Waist circumference was determined as the minimum circumference between the distal end of the rib cage and the top of the iliac crest along the midaxillary line.

#### 2.3.3. Blood Parameter

After an overnight fast, blood was sampled in the morning (7:00 to 9:00) in a sitting position from an antecubital vein. Serum samples were centrifuged at 3000 RPM for 20 minutes and analyzed by the “Zentrallabor” of the Medical Department I University of Erlangen-Nuremberg. Glucose, total cholesterol, HDL- and LDL cholesterol, and triglycerides (Olympus Diagnostica GmbH, Hamburg, Germany) were determined.

Blood pressure was determined in a sitting position after 5 minutes rest with an automatic oscillometric device (Bosco, Bosch, Jungingen, Germany). Measurements were taken in a nonfasting condition. Subjects refrained from coffee or tea for at least two hours prior to testing. 

#### 2.3.4. Questionnaires

To adequately assess physical activity and exercise at baseline and during the intervention, a questionnaire specifically developed to assess physical activity and exercise with impact on bone in this cohort was used [16]. Follow-up questionnaires additionally asked for corresponding changes during the intervention period, in particular with respect to diseases and medication, additional sport activities, and changes of physical activity and dietary intake. The good reproducibility of the questionnaires had been determined in an earlier stage of the study [15, 17]. 

In order to control nutritional changes, nutritional behavior was assessed using 5-day dietary protocols. The analysis of the protocols was performed using Prodi-4.5/03 Expert software (Wissenschaftlicher Verlag, Freiburg, Germany). 

#### 2.3.5. 10-Year CHD Risk and 10-Year Hard CHD Risk

Parameters constituting 10-year CHD risk in women [8] were age, diabetes status, smoking status, total cholesterol categories, LDL-C categories, and blood pressure categories while specific risk factors for 10-year hard CHD risk (myocardial infarction or cardiac death [10]) were age, total cholesterol, HDL-C, treatment for hypertension, and smoking status.

Based on score sheets, the corresponding 10-year risk (CHD risk/hard CHD risk) for each subject was given as a percentage value. 

#### 2.3.6. Metabolic Syndrome *Z*-Score

MetS *Z*-score was calculated according to the formula proposed by Johnson et al. [9] based on the NCEP-ATP III Criteria of the MetS. According to this criteria the MetS is prevalent if three out of the five risk factors were present: (1) raised triglyceride (TriGly) levels (≥150 mg/dL); (2) reduced HDL-C (<50 mg/dL for females, or specific treatment for previously detected hypertriglyceridaemia/reduced HDL-C); (3) raised blood pressure (≥130/85 mmHG, or specific treatment); (4) raised fasting plasma glucose (≥100 mg/dL); (5) waist circumference (WC > 88 cm for females).

According to Johnson et al. [9] for each parameter (i.e., HDL-C, Triglycerides) of the individual data, the ATP-III cut-point for a female population and the corresponding baseline standard deviation (SD) of the entire EFOPS-cohort were used. In detail, the *Z*-score was calculated: [(50 – HDL-C)/ SD HDL-C] + [(TriGly − 180)/ SD TriGly] + [(Glucose − 100)/ SD Glucose] + [(WC − 88)/ SD WC] + [(Mean arterial pressure (MAP) − 100)/ SD MAP]. 

### 2.4. Statistical Procedures

The sample size calculation of the present study was based on “10-year CHD risk.” In order to detect a 50% difference (i.e., changes of CHD risk in the EG half as high compared with the CG) between exercisers and control, 40 subjects per group were required for a 5% error probability with 80% statistical power. A per protocol analysis (PPA) was performed that excluded all subjects who underwent therapy with medications that relevantly affected primary endpoints after study start. Additionally, an intention to treat (ITT) analysis including all the subjects independently of adherence to the protocol or lost to follow-up was performed for primary and secondary endpoints (10-year CHD risk; MetS *Z*-score; 10-year hard CHD risk) using the “last observation carried forward” (LOCF) principle. 

Baseline characteristics were reported as means with standard deviations. Between-group differences of parameters presented in Tables 1 and 3 were calculated using Mann-Whitney *U* tests. Primary and secondary endpoints were log-transformed to obtain normally distributed data required for the analysis of variance with repeated measurements. Between-group differences are given as absolute difference with 95% confidence intervals (Tables 2 and 3). Within-group differences were analyzed with paired *t*-tests (text). Effect sizes (ES) based on the absolute difference (±standard deviation) between baseline and 12-year follow-up in the EG and CG were calculated using Cohen's d. SPSS 18.0 (SPSS Inc, Chicago, IL) was used for all statistical procedures.

## 3. Results

Figure 1 gives the participant flow during the EFOPS study between 1998 and 2010. Briefly 59 subjects of the EG still exercised after 12 years, and 48 subjects of the nontraining control were willing to perform the 12-year follow-up. Looking at the 27 dropouts of the EG, 5 subjects cited study-related reasons for their withdrawal (too intensive: *n* = 2; too frequent: *n* = 3). Twelve reported quitting the program for occupational reasons, 5 subjects developed serious diseases (e.g., asthma, cancer), 4 women moved, and one participant died. 

Attendance rate was 73% for the group session and 36% (reported performance rate) for the home training session. Thus, on average, participants of the EG exercised with an exercise frequency of 2.2 sessions/week (range: 1.4 to 3.0 sessions/week) representing an average weekly exercise volume of 92 min/week (range: 72 to 137 min/week). During the *≈*600 participant years of exercise one hairline fracture of the os pubis, three strain traumas, and two muscle fiber ruptures were recorded.

Apart from changes of medication affecting primary endpoints which resulted in exclusion, no significant differences regarding changes of parameters that may impact study results (i.e., physical activity, additional exercise, diet, life style) were determined between EG and CG. 

Table 2 shows the results for primary and secondary study endpoints. Taken together, changes of 10-year CHD risk (EG: 2.65 ± 2.09%, *P* = 0.001 versus CG: 5,40 ± 3.30%, *P* = 0.001), MetS *Z*-score (EG: −0.42 ± 1.03%, *P* = 0.003 versus CG: 1.61 ± 1.88%, *P* = 0.001), and 10-year risk of myocardial infarction/cardiac death (EG: 2.06 ± 1.17%; *P* = 0.001 versus CG: 3.26 ± 1.31%; *P* = 0.001) were significantly (all *P* = 0.001) more favorable in the EG compared with the CG. Ignoring the subjects' increasing age, which was, however, considered as a core risk factor by both CHD risk scores, changes of 10-year CHD risk and CHD risk were no more significantly negative in the EG, contrarily to the CG.

Since the per protocol analysis presented in Table 2 may provide too positive study effects, we also analyzed our data with the intention to treat principle using the last observation carried forward (OCF) method. Although the ITT-analysis presented in Table 3 resulted in slightly lower effect sizes, all the differences remained highly significant.

Thus, the hypothesis was clearly supported that changes of 10-year CHD risk, MetS *Z*-score, and 10-year risk of myocardial infarction/coronary death were significantly more favorable in the training group compared with nontraining controls.

With respect to the underlying mechanisms, Table 4 shows changes of modifiable, continuously scaled risk factors constituting metabolic and cardiac risk scores among the EG and CG. Briefly, significant negative changes (all *P* < 0.007) in the CG were determined for waist circumference (WC), RR-MAP, triglycerides, and total and LDL-cholesterol. In the EG significant negative changes were detected for WC and total cholesterol (both *P* = 0.001), while changes of RR-MAP, Glucose, and HDL-C were significantly positive. As given in Table 4, between-group differences with more favorable changes in the EG were assessed for WC, RR-MAP, triglycerides, LDL-C, and HDL-C.

Diabetes status (EG: *n* = 2 versus CG: *n* = 2) did not change during the study period; however, 3 out of 7 subjects of the EG stopped smoking and one exerciser markedly decreased her tobacco abuse, whereas the number of smokers in the CG (*n* = 5) increased by one subject.

## 4. Discussion

The central aim of the EFOPS study is to determine the comparative effects of increasing age/menopause versus regular exercise on health risk factors in women 1–8 years postmenopausal at baseline. In this contribution, the authors clearly demonstrated the positive long-term effect of an ambulatory multipurpose exercise program on metabolic and cardiac risk factor scores. There are many exercise trials that assessed the effect of various types of exercise on metabolic and cardiac risk factors [18–21]. However, this study extended the existing data by establishing that high intensity, multipurpose exercise programs with reasonable training volume positively impact metabolic and cardiac risk scores in the critical (early) postmenopausal years persistently over 12 years. Of methodological importance, highly validated cardiac risk scores [8, 10] could be used that included factors (i.e., diabetes status, smoking) hard to influence within short-term interventions. 

With respect to the primary and secondary study endpoints, an apparent discrepancy between positive changes for MetS *Z*-score and negative developments for both CHD risk factors may prevent a proper interpretation of the results of the EG. However, taking into account that “age” is a highly weighted risk factor within the 10-year CHD/hard CHD risk score concept [8, 10], the clinically moderate but statistically highly significant CHD risk changes in the EG (Tables 2 and 3) can be largely attributed to the increased age of the females. 

Unfortunately, the short study duration of most exercise studies along with the paucity of studies that take 10-year CHD risk as a study endpoint prevents a distinct discussion with respect to the present literature. Regarding 10-year CHD risk, two studies focus on the effect of exercise on 10 y CHD risk in postmenopausal women with an intervention ≥12 months. The Senior Fitness and Prevention study (SEFIP) [22] prescribed a comparable multipurpose exercise program for 18 months with independent living females 65 years and older. Not strictly in line with the present finding, the authors reported significant positive changes in the EG (−2.0 ± 3.8%) and in the active CG (−1.2 ± 2.8%) with no significant between-group differences. However, beside the study duration and subject age, the main methodological difference between both studies is the implementation of an active CG that exercised 4 × 10 weeks during the SEFIP study course with one session of 60 min/week of (very) low exercise intensity. Of interest, the favorable changes of 10 y CHD risk in the SEFIP-CG are mainly based on significant decreases of diastolic (8 ± 6%) and systolic blood pressure (5 ± 4%). With respect to the exercise protocol and the baseline status (early-postmenopausal) most closely corresponding to the present study, the Training and Cimicifuga Racemosa (TRACE) study [23] did not show significant differences between the EG (+0.2 ± 1.9%, *P* = 0.603) and the CG (+1.1 ± 2.1%; *P* = 0.007) which also performed the low intensity/low volume exercise protocol described earlier. Although one may argue that the menopausal transition along with its hormonal and metabolic turbulences is a nonrepresentative phase in female life, this result could not be anticipated. Considering that the exercise protocol of the TRACE study focuses even more on CHD risk factors compared with the EFOPS exercise program, exercise effects on 10-year CHD risk were expected to be much more pronounced in the TRACE study. To which degree this result could be due to the different stages of the menopausal transition (TRACE: 1–3 versus EFOPS: 1–8 year postmenopausal) is debatable. However, in summary, readers and researchers should notice that the effect of identical (CG: SEFIP versus Trace) or comparable (EG: SEFIP versus Trace versus EFOPS) exercise protocols on CHD risk profoundly varied although the female cohorts were quite homogeneous concerning age, menopausal status, and physical fitness.

Contrary to the more static 10-year CHD risk score, many more exercise trials focus on MetS prevalence (i.e., [24–29]), number of MetS risk factors (i.e., [9, 30, 31]), or continuously scaled MetS scores [9, 32]. With respect to the latter parameter, the results of this study largely confirmed the positive results of two studies [9, 32], which included postmenopausal female cohorts (40–65 y) with elevated LDL-C or decreased HDL-C-levels, however. Although the isolated endurance exercise programs of Camhi et al. (3 × 45–60 min/week aerobic exercise at 60–85% HRmax for 12 months) and Johnson et al. (aerobic exercise: 179 min/week at 40%–55% VO_2_ peak versus 114 min at 65%–80% VO_2_ peak versus 175 min at 65%–80% VO_2_ peak for 8 months) widely differ from the present exercise protocol, in line with our data, 3 out of 4 exercise protocols/study arms resulted in significant positive MetS *Z*-score changes among the EG and significant differences compared with a nontraining control group (with slight, nonsignificant changes) while results of one study arm (114 min at 65%–80% VO_2_ peak; [9]) did not reach statistical significance. 

With respect to the underlying mechanisms, changes of isolated risk factors constituting 10-year CHD/hard CHD risk and MetS according to IDF [33] or NCEP ATP III [10] were analyzed in the present study. As the main result of this analysis, changes among the EG were far from uniform with significant negative changes for waist circumference (WC) and total cholesterol and significant positive changes for RR-MAP, Glucose, and HDL-C. Most interestingly, although it is frequently reported particularly for female cohorts that WC and CHD risk is closely related [34, 35] and some authors [32, 34] attributed positive changes of CHD risk and MetS status to corresponding changes of WC, the present result does not support a high longitudinal correlation of both factors. However, despite the general cross-sectional validity of waist circumference to represent visceral adipose tissue (VAT) [36, 37], exercise induced changes of visceral fat as assessed by MRT/CT were not relevantly reflected by changes of WC [18]. On the other hand, it is hard to believe that WC increments of almost 10% in the EFOPS-EG were not due to relevantly raised VAT. 

Judging the relevance of the present study, strength and limitations of EFOPS must be adequately considered. Starting with the strong points, the central facet of this study is its closely supervised exercise program for 12 years so far. To derive adequate exercise recommendations and robust results, trial interventional period should at least exceed the period of initial adaptation to exercise. Revisiting basic exercise principles, strains that initially increase system thresholds may become ineffective after adaptation of the system addressed [38, 39]. Translated into clinical practice this suggested that even when protocols are effective during the first months of exercise, this does not guarantee general effectiveness. Further strong points were (a) a homogenous group of (early) postmenopausal females, for which CHD risk is increasingly relevant. (b) The exercise protocol was progressively increased and regularly adjusted to subjects' capability during the entire intervention period. (c) Group sessions were closely supervised by certified trainers. (d) Covariates (diseases, medication, nutrition, life style) affecting study endpoints were strictly requested and controlled throughout the study in order to exclusively relate results to the given intervention (see also limitations). (e) Low drop-out rates and high overall attendance/compliance rates (at least with respect to the study length of 12 years so far) [40] indicate the attractiveness of the exercise program. (f) Two different methods of analysis (PPA and ITT) resulted in congruent results indicating the high reliability of our findings.

However, a number of limitations decrease the evidence of the study: (a) from a strictly methodological/statistical point of view, the nonrandomized group allocation may considerably reduce the evidence of our study results. We were aware of this problem; however, with respect to the intended long study duration, three main arguments encouraged our decision to allow the participants to join the study arm of their choice. (1) Though negligible in short-term studies, study allocation to the “wrong” (i.e., undesired) study arm of (very) long-term studies may produce a severe bias with respect to adherence. The most serious risk is that subjects who are willing to exercise but are randomized into the (life-long) inactive CG would exercise on their own without disclosing the fact and thus have a negative impact on the validity of the findings. (2) With respect to dropout and loss to follow-up, randomization into the (wrong) study will relevantly increase withdrawal of the subjects. Besides the loss of statistical power and a subsequent attrition bias, the fact that drop-out rates higher than 20% for short-term and 30% for long-term studies may break the random assignment to the study arms [41]. (3) In order to provide a realistic insight into long-term compliance and attendance of motivated participants, we decided to dispense with randomization. With respect to the issue of whether our results are generalizable to “random” osteopenic females, from a pragmatic point of view this question may be inappropriate and irrelevant since the majority of these females would never perform an intense 12-year exercise program, thus making any comparison impossible.

(b) Despite consistent monitoring of confounders, it cannot be claimed that all the determinants during these 12 years were perfectly controlled. This may be especially true changes of physical activity. Although a great deal of effort went into monitoring physical activity, our physical activity questionnaires [15, 16] are “bone specific,” and, hence, it may not have picked up slight changes of physical activities that impact the metabolic and/or cardiac system. 

(c) Due to the nonsupervised home exercise program, exercise frequency may not be perfectly recorded for this component of the exercise training.

## 5. Conclusion

Although the nonrandomized design may prevent a definite conclusion, in summary EFOPS provides a large body of evidence that multipurpose exercise programs significantly and consistently address relevant (early) postmenopausal risk factors as determined for CHD risk and bone loss/fracture risk [12], at least for the minority of postmenopausal subjects willing to exercise [42].

## Figures and Tables

**Table 1:** Baseline characteristics of the exercise and the control groups.

Variable	Exercise EG (*n* = 51)	Control CG (*n* = 43)
Age (years)	54.8 ± 3.6	55.8 ± 3.2
Body mass index (kg/m^2^)	24.8 ± 3.0	25.6 ± 3.6
Body fat (%)^b^	35.8 ± 4.6	35.2 ± 5.8
Age at menarche (years)^a^	13.1 ± 1.4	13.3 ± 1.6
Age at menopause (years)^a^	50.6 ± 3.1	50.6 ± 3.3
Volume of exercise (min/week)^a^	89 ± 84	75 ± 66
Physical activity (Index)^a,c^	4.1 ± 1.2	4.0 ± 1.3
VO_2_ peak (mL/min/kg)^d^	25.3 ± 6.3	25.3 ± 5.9
Energy intake (MJ/d)^e^	7.75 ± 1.39	7.69 ± 1.85
Fat intake (% of energy intake)^e^	35 ± 8	36 ± 7
Smokers (%)^a^	14	12
Diabetes (%)^a^	4	5
Prevalence metabolic syndrome (%)^f^	12	12

No significant between-group differences were determined. ^a^As determined by questionnaire; ^b^as determined by bioimpedance analysis (Tanita BF 305, Tokyo, Japan); ^c^as determined by physical activity questionnaire (1: very low to 7: very high) [15], ^d^stepwise treadmill test to voluntary maximum; ^e^5-day dietary analysis (Prodi-4.5/03, Wissenschaftlicher Verlag, Freiburg, Germany); ^f^according to NCEP-ATP III.

**Table 2:** Per protocol analysis: changes in the exercise and control groups for primary and secondary study endpoints.

	Exercise MV (SD) *n* = 51	Control MV (SD) *n* = 43	Absolute difference MV (95% CI)	*P *	Effect size (*d*)
10-year CHD risk					
Baseline (%)	8.39 ± 3.27	7.42 ± 2.58	—	—	—
12 y follow-up (%)	11.04 ± 3.16	12.81 ± 3.08	3.89	—	—
Risk changes (%)	2.65 ± 2.09***	5.40 ± 3.30***	2.75 (1.61 to 3.89)	0.001	0.90

Metabolic syndrome *Z*-score					
Baseline (%)	−2.44 ± 2.39	−3.65 ± 2.84	—	—	—
12 y follow-up (%)	−2.88 ± 3.01	−2.04 ± 3.77	—	—	—
Changes (%)	−0.42 ± 1.03**	1.61 ± 1.88***	2.03 (1.42 to 2.64)	0.001	1.36

10-year myocardial infarction/cardiac death risk (hard 10-year CHD risk)					
Baseline (%)	1.78 ± 1.44	1.60 ± 1.26	—	—	—
12 y follow-up (%)	3.84 ± 1.41	4.86 ± 2.01	—	—	—
Risk changes (%)	2.06 ± 1.17***	3.26 ± 1.31***	1.20 (0.68 to 1.71)	0.001	0.97

Significance (*P*) for within-group effects: **P < 0.01, ***P < 0.001. Exact significance values are listed in the Result Section.

**Table 3:** Intention to treat analysis: changes in the exercise and control groups for primary and secondary study endpoints.

	Exercise MV (SD) *n* = 86	Control MV (SD) *n* = 51	Absolute difference MV (95% CI)	*P *	Effect size (*d*)
10-year CHD risk					
Baseline (%)	8.42 ± 3.14	7.49 ± 2.72	—	—	—
12 y follow-up (%)	10.24 ± 3.22	11.69 ± 2.80	3.89	—	—
Risk changes (%)	1.80 ± 2.16***	4.23 ± 3.10***	2.44 (1.55 to 3.33)	0.001	0.91

Metabolic syndrome *Z*-score					
Baseline (%)	−2.47 ± 2.39	−3.54 ± 2.60	—	—	—
12 y follow-up (%)	−2.72 ± 3.09	−2.07 ± 3.44	—	—	—
Changes (%)	−0.25 ± 1.44^n.s.^	1.47 ± 1.76***	1.72 (1.17 to 2.27)	0.001	1.07

10-year myocardial infarction/cardiac death risk (hard 10-year CHD risk)					
Baseline (%)	1.71 ± 1.11	1.62 ± 1.20	—	—	—
12 y follow-up (%)	3.02 ± 1.56	4.18 ± 1.61	—	—	—
Risk changes (%)	1.31 ± 1.35***	2.55 ± 1.40***	1.23 (0.76 to 1.72)	0.001	0.90

Significance (*P*) for within-group effects: ***P < 0.001; n.s.: nonsignificant.

**Table 4:** Changes of modifiable CHD risk factors constituting the metabolic and cardiac risk scores.

Parameter	Exercise MV (SD) *n* = 51	Control MV (SD) *n* = 43	Absolute difference MV (95% CI)	*P *	Effect size (*d*)
Waist circumference (cm)	7.43 ± 5.42	11.33 ± 5.26	3.90 (1.70 to 6.09)	0.001	0.73
RR-MAP (mmHg)	−3.22 ± 5.11	4.65 ± 6.31	7.87 (5.53 to 10.21)	0.001	1.37
Glucose (mg/dL)	−2.80 ± 6.40	0.09 ± 7.89	2.90 (−0.03 to 5.82)	0.052	0.40
Triglycerides (mg/dL)	−0.1 ± 22.7	10.9 ± 24.9	11.1 (1.34 to 20.85)	0.026	0.46
Total cholesterol (mg/dL)	11.9 ± 22.1	18.5 ± 22.2	6.6 (3.9 to 27.5)	0.157	0.30
HDL-cholesterol (mg/dL)	7.37 ± 6.96	1.07 ± 8.22	6.30 (2.3 to 10.30)	0.001	0.83
